# Thermoelectric Performance of Tetrahedrite (Cu_12_Sb_4_S_13_) Thin Films: The Influence of the Substrate and Interlayer

**DOI:** 10.1021/acsaelm.3c00909

**Published:** 2023-09-25

**Authors:** Yu Liu, Andrey V. Kretinin, Xiaodong Liu, Weichen Xiao, David J. Lewis, Robert Freer

**Affiliations:** † Department of Materials, University of Manchester, Oxford Road, Manchester M13 9PL, U.K.; ‡ National Graphene Institute, University of Manchester, Oxford Road, Manchester M13 9PL, U.K.

**Keywords:** thermoelectric, thin film, tetrahedrite Cu_12_Sb_4_S_13_, Sb_2_O_3_, buffer layer, substrate

## Abstract

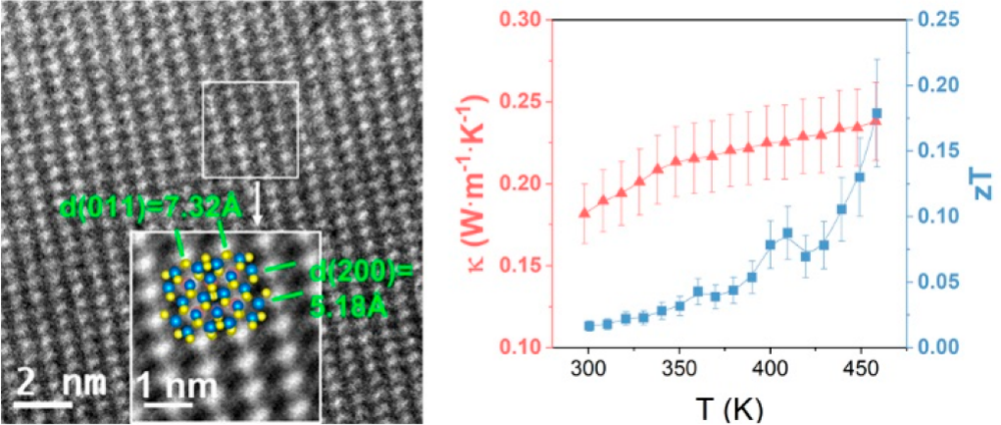

In the present work, tetrahedrite Cu_12_Sb_4_S_13_ thin films were deposited on various substrates via aerosol-assisted chemical vapor deposition (AACVD) using diethyldithiocarbamate complexes as precursors. A buffer layer of Sb_2_O_3_ with a small lattice mismatch to Cu_12_Sb_4_S_13_ was applied to one of the glass substrates to improve the quality of the deposited thin film. The buffer layer increased the coverage of the Cu_12_Sb_4_S_13_ thin film, resulting in improved electrical transport properties. The growth of the Cu_12_Sb_4_S_13_ thin films on the other substrates, including ITO-coated glass, a SiO_2_-coated Si wafer, and mica, was also investigated. Compared to the films grown on the other substrates, the Cu_12_Sb_4_S_13_ thin film deposited on the SiO_2_-coated Si wafer showed a dense and compact microstructure and a larger grain size (qualities that are beneficial for carrier transport), yielding a champion power factor (PF) of ∼362 μW cm^–1^ K^–2^ at 625 K. The choice of substrate strongly influenced the composition, microstructure, and electrical transport properties of the deposited Cu_12_Sb_4_S_13_ thin film. At 460 K, the highest *zT* value that was obtained for the thin films was ∼0.18. This is comparable to values reported for Cu–Sb–S bulk materials at the same temperature. Cu_12_Sb_4_S_13_ thin films deposited using AACVD are promising for thermoelectric applications. To the best of our knowledge, the first full thermoelectric characterization of the Cu_12_Sb_4_S_13_ thin film is performed in this work.

## Introduction

In recent decades, thermoelectric materials have been extensively investigated in response to the challenges of the energy transition driven by global warming. The burning of fossil fuels, a primary source of power generation, releases both heat and greenhouse gases into the environment.^1^ However, this heat energy waste can be harvested and converted into electrical power by thermoelectric materials without gaseous emissions or fuel consumption.^2^ The conversion efficiency of a thermoelectric material is related to the thermoelectric figure of merit *zT*, which is defined as^3^
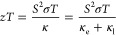
1where *S* is the Seebeck coefficient, σ is the electrical conductivity, *T* is the absolute temperature, κ is the thermal conductivity, *κ*_e_ is the electronic thermal conductivity, and *κ*_l_ is the lattice thermal conductivity. To achieve a high conversion efficiency, a thermoelectric material should possess a large Seebeck coefficient and high electrical conductivity but low thermal conductivity.

The Cu–Sb–S system includes four main phases (Figure S1): tetrahedrite (Cu_12_Sb_4_S_13_), famatinite (Cu_3_SbS_4_), skinnerite (Cu_3_SbS_3_), and chalcostibite (CuSbS_2_).^4^ Among these phases, tetrahedrite Cu_12_Sb_4_S_13_ is widely studied for thermoelectric applications due to its intrinsically low thermal conductivity.^5,6^ Tetrahedrite Cu_12_Sb_4_S_13_ has a space group of *I*43*m*; its unit cell contains 58 atoms, and there are five crystallographically distinct atomic sites: Cu(1), Cu(2), S(1), S(2), and Sb. These sites form three types of structural units: Cu(1)S_3_ tetrahedra, Cu(2)S_3_ trigonal planes, and SbS_3_ trigonal pyramids. A strong lattice anharmonicity arising from atomically displaced Cu(2) atoms and Sb lone-pair electrons suppresses the lattice thermal conductivity and boosts *zT*.^7^ Combining this with the inherent advantages of sulfides, such as low toxicity, earth abundance, and low cost, makes Cu_12_Sb_4_S_13_ a promising candidate for thermoelectric applications.

Since Suekuni et al. reported the thermoelectric properties of Cu_12_Sb_4_S_13_ for the first time in 2012,^5,8^ pure Cu_12_Sb_4_S_13_ bulk materials have been fabricated by various techniques to improve their thermoelectric performance. Wang et al. synthesized Cu_12_Sb_4_S_13_ ingots by melting and subsequent annealing, obtaining a main phase of Cu_12_Sb_4_S_13_ with two impurity phases, Cu_3_SbS_4_ and CuSbS_2_. A maximum *zT* value of 0.52 at 600 K was reported for samples annealed at 723 K for 24 h.^9^ Sun et al. reported a maximum *zT* of 0.65 at 723 K for Cu_12_Sb_4_S_12.7_ prepared by mechanical alloying (MA) and spark plasma sintering (SPS),^10^ while Kim et al. combined MA and hot pressing (HP) to prepare Cu_12_Sb_4_S_13_ with a secondary phase of Cu_3_SbS_4_ and a considerably improved maximum *zT* of 0.87 at 723 K.^11^ Yang et al. reported hot-pressed Cu_11.8_Sb_4.2_S_13_ with an impurity phase of CuSbS_2_, which had a maximum *zT* of 0.8 at 700 K.^12^ Lu et al. reported that the thermoelectric performance of the material was further improved through doping, and the maximum *zT* at 723 K reached ∼1.03 for Ni and Zn co-doped Cu_10.5_NiZn_0.5_Sb_4_S_13_.^13^ Another relatively high *zT* value of ∼1.1 at 723 K was reported by Yan et al. for Cu_13.5_Sb_4_S_12_Se.^14^

Thin-film thermoelectric materials offer numerous advantages, including low costs, low weights, and niche deployment opportunities. These qualities mean that thin-film materials are particularly well-suited for the development of compact devices, such as wearable electronics and microscale cooling systems, in contrast to the challenges bulk materials face in small-scale applications.^15^ In view of the excellent thermoelectric performance of Cu_12_Sb_4_S_13_ bulk materials, there should be opportunities for Cu_12_Sb_4_S_13_ thin films to be used in portable and wearable thermoelectric devices. Kumar et al. demonstrated the synthesis of Cu_12_Sb_4_S_13_ thin films via electron beam evaporation, yielding a maximum power factor (PF, *S*^2^σ) of 2.30 μW cm^–1^ K^–2^ at 495 K.^16^ However, thermoelectric effects in Cu_12_Sb_4_S_13_-based thin films have not yet been reported, which motivates us to investigate the potential of Cu_12_Sb_4_S_13_ thin films in thermoelectric applications.

Aerosol-assisted chemical vapor deposition (AACVD) (Figure S2), is a variant of CVD, which allows for the uniform deposition of thin films on substrates but at ambient pressure.^17^ Makin et al. synthesized Cu–Sb–S thin films via AACVD using different ratios of bis(diethyldithiocarbamate)copper(II) [Cu(S_2_CN(C_2_H_5_)_2_)_2_] and tris(diethyldithiocarbamate)antimony(III) [Sb(S_2_CN(C_2_H_5_)_2_)_3_], and obtained Cu_12_Sb_4_S_13_ thin films when the [Cu]/([Cu] + [Sb]) precursor ratio was 0.6 and 0.8.^18^

Given the potential widespread utilization of thin films in thermoelectric devices, it is essential to understand the influence of candidate substrates on the quality of the thin film, as well as how they control thermoelectric properties.^19^ The lattice structure of the substrate could affect the orientation of the thin film, and any lattice mismatch between the thin film and the substrate will cause strain and defects in the thin film.^20,21^ The difference between the thermal expansion coefficients of the thin film and the substrate can induce thermal stress, potentially causing cracking or delamination of the film from the substrate.^22^ For these reasons, selecting substrates with similar lattice constants and thermal expansion coefficients to those of the target thin-film material is important. In a number of thermoelectric thin-film studies, an intermediate buffer layer with a comparable lattice constant and/or thermal expansion coefficient has been applied to the substrate prior to deposition to reduce mismatch and improve the quality of the thin film. Moreover, an intermediate layer can also act as a seed layer to improve the nucleation efficiency.^23^ In the case of InGaZnO thin films grown by Seo et al., the use of a ZnO buffer layer significantly reduced lattice mismatch, leading to an enhancement of PF from ∼1.5 to ∼18 μW m^–1^ K^–2^ at 375 K.^20^ Similarly, Zhou et al. obtained high-quality Ga-doped ZnO thin films on *c*-sapphire substrates with a 10 nm ZnO buffer layer, resulting in an improved PF of 449 μW m^–1^ K^–2^ at 623 K.^24^ The quality of the thin films is also related, to some degree, to the roughness, hydrophilicity, and morphology of the substrate surface.^25^

In the present work, tetrahedrite Cu_12_Sb_4_S_13_ thin films were deposited on amorphous soda-lime glass substrates (hereafter referred to as glass substrates) via AACVD using Cu(S_2_CN(C_2_H_5_)_2_)_2_ and Sb(S_2_CN(C_2_H_5_)_2_)_3_ as precursors. To improve the quality of the thin films, an Sb_2_O_3_ buffer layer was applied between the Cu_12_Sb_4_S_13_ thin film and the glass substrate. Other substrates, including indium tin oxide (ITO)-coated glass, a SiO_2_-coated Si wafer, and mica, were also employed to investigate the effects of the different substrates on the composition, microstructure, and electrical transport properties of the Cu_12_Sb_4_S_13_ thin films. A complete thermoelectric characterization of the Cu_12_Sb_4_S_13_ thin films was carried out on an instrument-dedicated Si_3_N_4_ test chip, yielding a maximum *zT* value of ∼0.18 at 460 K.

## Experimental Section

Sb_2_O_3_ buffer layers were applied to glass substrates (hereafter referred to as Sb_2_O_3_-coated glass) by depositing an Sb_2_S_3_ thin film and then annealing in air at 450 °C for 60 min. In light of an earlier investigation, where stable and reliable Sb_2_S_3_ films were prepared from tris(*O*-ethylxanthato)antimony(III) [Sb(S_2_COC_2_H_5_)_3_], this same precursor was selected in the present work for the deposition of Sb_2_S_3_ thin films using AACVD.^26^ Then, tetrahedrite Cu_12_Sb_4_S_13_ thin films were deposited on the substrates via AACVD using Cu(S_2_CN(C_2_H_5_)_2_)_2_ and Sb(S_2_CN(C_2_H_5_)_2_)_3_ as precursors, based on a previous work by Makin et al.^18^

### Synthesis and Characterization of Precursors

The precursors were typically prepared under a dry nitrogen (N_2_) atmosphere using a Schlenk line. All reagents were obtained from Sigma-Aldrich and used without further purification. The synthesis of the precursors is described in detail in the Supporting Information. Elemental analysis was conducted by the microanalytical laboratory at the University of Manchester. C, H, and N microanalyses were carried out using a Thermo Fisher FlashSmart CHNS/O elemental analyzer; S content was determined with a Thermo Fisher Flash 2000 CHNS/O analyzer, and the metal analysis was conducted by inductively coupled plasma atomic emission spectroscopy (ICP-AES) using a Thermo Fisher iCAP 6300 DUO system. Thermogravimetric analysis (TGA) and differential scanning calorimetry (DSC) were carried out under a N_2_ atmosphere using a Mettler Toledo TGA/DSC 1 system in the temperature range 30–500 °C and with a ramp rate of 10 °C min^–1^. The TGA and DSC data for the precursors are presented in Figure S3.

### Deposition of Tetrahedrite Cu_12_Sb_4_S_13_ Thin Films

The typical AACVD procedure was as follows. First, the precursors were dissolved in a solvent (exact details to follow). The solution was transferred to a 100 mL two-neck flask, which was connected to a glass tube placed in a Carbolite furnace (to act as a hot-wall reactor); the other side of the two-neck flask was connected to an argon (Ar) gas supply. All of the substrates were cleaned by ultrasonication in 30 mL of acetone for 30 min. Then, they were dried by compressed N_2_ gas and loaded into the glass tube. A Maplin digital ultrasonic humidifier was placed underneath the two-neck flask containing the solution to generate solution aerosol. The Ar gas was used as a carrier to transport the aerosol droplets to the hot-wall reactor, where, with the evaporation of the solvent and the decomposition of the precursors, the depositing material reacted with the substrate surface to generate the thin films.

#### Deposition of Sb_2_O_3_ Buffer Layer on Glass Substrate

The precursor Sb(S_2_COC_2_H_5_)_3_ (0.3 g, 0.62 mmol) was dissolved in 20 mL of tetrahydrofuran (THF) as the AACVD deposition solution. The glass substrates were cleaned and placed in the hot-wall reactor. An Ar flow rate of 200 cm^3^ min^–1^ was employed, and the deposition temperature was 250 °C. After 60 min of deposition, the obtained thin film was annealed in air at 450 °C for 60 min to ensure that the thin film was fully oxidized to Sb_2_O_3_. The obtained Sb_2_O_3_-coated glass was directly utilized as a substrate for the deposition of the Cu_12_Sb_4_S_13_ thin film without extra cleaning.

#### Deposition of Cu_12_Sb_4_S_13_ Thin Films on Different Substrates

The AACVD deposition parameters for the Cu_12_Sb_4_S_13_ thin films in this work are primarily adapted from the earlier work of Makin et al.^18^ The deposition solution was prepared by the mixing precursors Cu(S_2_CN(C_2_H_5_)_2_)_2_ (0.20 g, 0.55 mmol) and Sb(S_2_CN(C_2_H_5_)_2_)_3_ (0.20 g, 0.35 mmol) in 30 mL of THF solvent with a [Cu]/([Cu] + [Sb]) ratio of 0.6. The flow rate for the Ar gas was 160 cm^3^ min^–1^. The deposition temperature of the hot-wall reactor was 450 °C, and the deposition time was 90 min. The as-deposited thin films were cooled to room temperature under flowing Ar. The following substrates were the ones that were used for the deposition of the Cu_12_Sb_4_S_13_ thin films: the prepared Sb_2_O_3_-coated glass, commercially obtained normal glass (Fisherbrand), ITO-coated glass (Ossila), a SiO_2_-coated Si wafer (Ossila), and mica (Sigma-Aldrich).

The lattice mismatch (*f*) between a thin film material and a substrate is expressed by eq 2:^27^
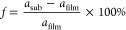
2where *a*_sub_ and *a*_film_ are the lattice parameters of the substrate and thin film, respectively. The lattice mismatch *f* between Cu_12_Sb_4_S_13_ and the substrates was calculated using eq 2. The root-mean-square (rms) roughness for the commercial glass slide, the SiO_2_-coated Si wafer, and the mica were taken from measured values in previous works;^28−30^ the electrical conductivity of the SiO_2_-coated Si wafer and ITO-coated glass and the rms roughness for the commercial ITO-coated glass were taken from the manufacturer’s data.^31^ Detailed information about the roughness of the substrates is listed in Table S1.

### Characterization of Tetrahedrite Cu_12_Sb_4_S_13_ Thin Films

Grazing incidence X-ray diffraction (GIXRD) was carried out on the substrates and thin films using a PANalytical X’Pert Pro diffractometer with a Cu Kα source (λ = 1.540598 Å); the angle of incidence was 3°. The XRD patterns of the thin films were indexed using the X’Pert Highscore Plus software, and the phases were identified with the TOPAS software^32^ and Rietveld refinement.^33,34^ The degree of preferred orientation in the films was evaluated from the XRD data in terms of the Lotgering factor (LF) via eq 3:^35^
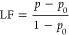
3where , ,  is the sum of the peak intensities of the standard data, and  is the sum of the intensities of the measured data. LF varies between 0 and 1, where LF = 0 corresponds to random orientation and LF = 1 corresponds to perfect orientation along a preferred growth direction.

The microstructures and compositions of the films were investigated by scanning electron microscopy (SEM) using a TESCAN MIRA3 SC system equipped with energy dispersive X-ray spectroscopy (EDX). The grain sizes and their distributions in the thin films were determined from SEM images using the ImageJ software. High-resolution transmission electron microscopy (HRTEM) images and selected area electron diffraction (SAED) patterns were obtained using an FEI Tecnai 20 TEM instrument operating at 200 kV.

Raman spectra and optical absorption spectra were collected with a Horiba LabRAM HR Evolution spectrometer and a PerkinElmer Lambda 1050 UV/Vis/NIR spectrophotometer, respectively. The optical bandgap was estimated from the optical absorption spectra using Tauc plots.

The in-plane Seebeck coefficients, electrical conductivities, and power factors of the Cu_12_Sb_4_S_13_ thin films on the different substrates were obtained using a ULVAC-RIKO ZEM-3 system under a low-pressure helium (He) atmosphere (approximately −0.09 MPa) at temperatures in the range 325–675 K. A complete thermoelectric characterization of the Cu_12_Sb_4_S_13_ thin films in the temperature range 300–450 K was performed with a Linseis thin film analyzer (TFA)^36,37^ under vacuum. For the TFA measurements, a Cu_12_Sb_4_S_13_ thin film was deposited on a TFA test chip with a 300 nm instrument-dedicated Si_3_N_4_ membrane using the same AACVD parameters as those used for the thin film deposition on the different substrates. Detailed procedures for the TFA measurements are described in our earlier work.^15^

## Results and Discussion

### Compositional and Structural Analysis

**Figure 1:**
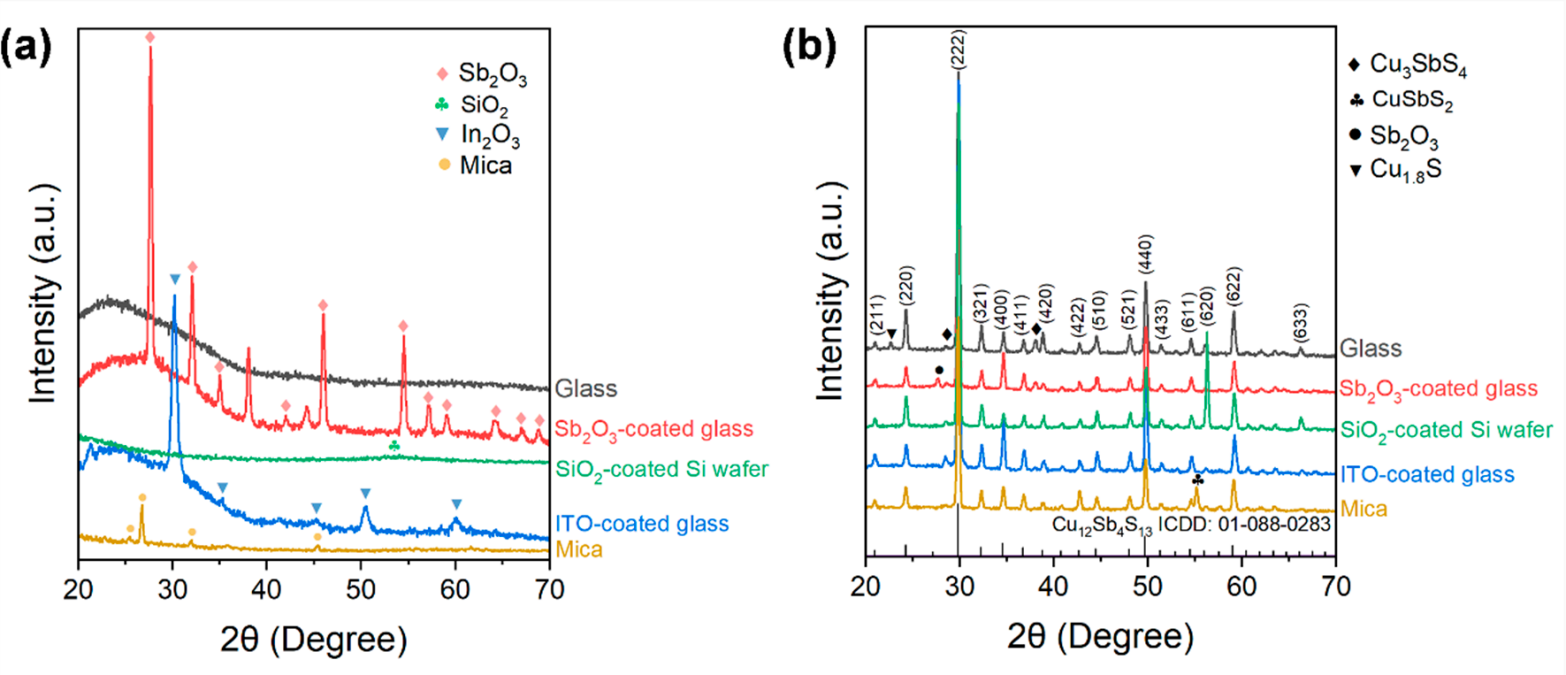
Grazing incidence X-ray diffraction (GIXRD) patterns for (a) the different substrate materials (glass, Sb_2_O_3_-coated glass, ITO-coated glass, a SiO_2_-coated Si wafer, and mica) and (b) the Cu_12_Sb_4_S_13_ thin films on these substrates.

Figure 1a shows the XRD patterns for the materials that were used as substrates (glass, Sb_2_O_3_-coated glass, ITO-coated glass, a SiO_2_-coated Si wafer, and mica). Compared to the XRD pattern for the glass substrate, the sharp reflections observed in the pattern for the Sb_2_O_3_-coated glass indicate that a crystallized Sb_2_O_3_ buffer layer was successfully deposited. An SEM image of the Sb_2_O_3_ buffer layer is shown in Figure S4. The broad reflections seen in the pattern for the SiO_2_-coated Si wafer could be due to ultrafine SiO_2_ surface grains. For the ITO-coated glass, the presence of ITO on the glass surface is also confirmed by its XRD pattern. Additionally, the XRD pattern for mica is consistent with previously published data, confirming that the cleavage plane is along the (001) plane.^38^

Optical images showing the gross appearance of the Cu_12_Sb_4_S_13_ thin films deposited on the glass, Sb_2_O_3_-coated glass, ITO-coated glass, SiO_2_-coated Si wafer, and mica substrates are presented in Figure S5. The variation in the coverage of the thin films can be related directly to lattice mismatch. In terms of the uniformity of coverage, the best films were achieved on the ITO-coated and Sb_2_O_3_-coated substrates, where the lattice mismatch values were 2.7% and 7.5%, respectively (Table S1). In contrast, the coverage of the films on the mica and SiO_2_-coated Si wafer substrates was poorer, as their values of lattice mismatch were higher (Table S1). There was also poor coverage on the amorphous glass substrates. The extent of coverage of the thin film has a significant impact on carrier transport (vide infra).

XRD patterns for the Cu_12_Sb_4_S_13_ thin films deposited on the different substrates are presented in Figure 1b. The main reflections in the XRD patterns can be indexed to cubic tetrahedrite Cu_12_Sb_4_S_13_ (ICDD: 01-088-0283), with impurity peaks belonging to Cu_1.8_S, Cu_3_SbS_4_, and CuSbS_2_; the latter can be explained by their closeness in stoichiometry to Cu_12_Sb_4_S_13_ (see the phase diagram in Figure S1). These impurity phases correspond to those reported previously for Cu_12_Sb_4_S_13_ materials.^9,12^

**Figure 2:**
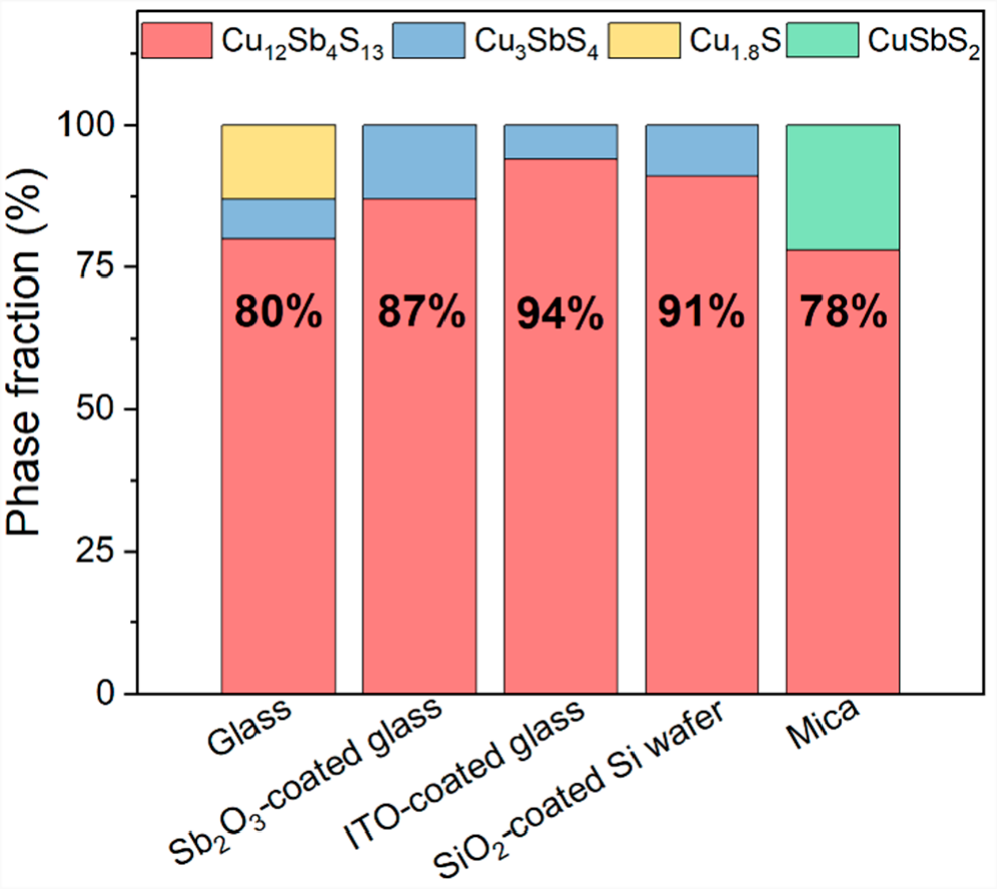
Content fractions of the phases in the thin films deposited on the following different substrates: glass, Sb_2_O_3_-coated glass, ITO-coated glass, a SiO_2_-coated Si wafer, and mica.

Figure 2 summarizes the phase content of the thin films deposited on the different substrates determined by Rietveld refinement. It is interesting to note that the thin film deposited on the glass substrate contains two impurity phases (Cu_3_SbS_4_ and Cu_1.8_S, together equivalent to 20%), while all of the other films contain only a single impurity phase. The use of a buffer or coating layer appears to lead to higher levels of the primary phase (∼90%), and all three of the coated substrates (Sb_2_O_3_-coated glass, ITO-coated glass, and the SiO_2_-coated Si wafer) contain the same secondary phase (Cu_3_SbS_4_). Compared to the amorphous glass substrate with poor crystallinity, the Sb_2_O_3_-coated glass, ITO-coated glass, and SiO_2_-coated Si wafer substrates appear to be advantageous for the growth of the main phase. In contrast, the films deposited on mica contain about 22% of a different secondary phase (CuSbS_2_). These impurity phases may affect the thermoelectric properties of the films due to there being a phase that has a different electrical conductivity than Cu_12_Sb_4_S_13_^12^ and through the scattering of carriers.

The Rietveld-refined lattice parameters for the Cu_12_Sb_4_S_13_ thin films on the different substrates are summarized in Table S2. There are only limited variations in the lattice parameters for Cu_12_Sb_4_S_13_ in these thin films, which suggests that the lattice mismatch between the thin films and the substrates could be compensated by structural distortions of the lattice.^22^ For example, the Cu_12_Sb_4_S_13_ thin film deposited on the ITO-coated glass substrate, which had the lowest lattice mismatch, exhibits the smallest lattice parameter of 10.3501(6) Å.

A comparison of the peak intensities of the thin films deposited on the different substrates (Figure 1b) in the XRD patterns reveals a relatively strong (620) peak for the thin film deposited on the SiO_2_-coated Si wafer and strong (2*kl*) peaks for the film deposited on the ITO-coated glass, indicating some degree of orientation in the films. The calculated Lotgering factors (LFs) for the (620) and (2*kl*) planes for the thin films are shown in Table S3. The thin film on the SiO_2_-coated Si wafer exhibits the highest LF for the (620) plane, and the thin film on the ITO-coated glass shows the largest LF for the (2*kl*) planes.

**Figure 3:**
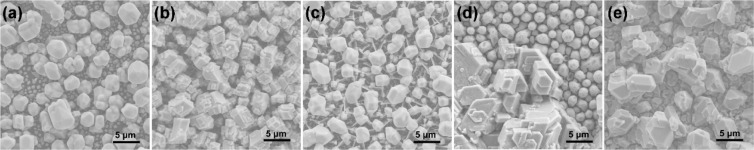
Scanning electron microscopy (SEM) images of the Cu_12_Sb_4_S_13_ thin films on the following different substrates: (a) glass, (b) Sb_2_O_3_-coated glass, (c) ITO-coated glass, (d) mica, and (e) a SiO_2_-coated Si wafer.

The morphologies and cross-sectional images of the thin films deposited on the various substrates are illustrated in Figures 3 and S7. The Cu_12_Sb_4_S_13_ thin film deposited on the glass substrate shows poor coverage and irregular grains having an average size of ∼0.37 μm. In contrast, the thin film on the Sb_2_O_3_-coated glass exhibits much better coverage and larger, uniform pseudocubic grains (∼1.08 μm). These results are consistent with the earlier simple coverage uniformity analysis (Figure S5). The improved quality of the latter thin film may be due to the fact that the Sb_2_O_3_ buffer layer has a similar lattice parameter to that of Cu_12_Sb_4_S_13_, thereby inducing a more ordered thin-film growth.^39^ This is reflected with a larger LF value (Table S3). The Sb_2_O_3_ layer could also act as a seed layer that assists nucleation and more uniform growth.^23^ The thin film on the ITO-coated glass shows spherical-like grains; interestingly, however, there are also small flake-shaped grains standing vertically on the substrate surface underneath the larger grains. This localized texturing gives rise to the larger-than-average LF value for this film (Table S3). In contrast, for the thin film deposited on mica, there is a mixture of uniform, small-sized spherical grains and discontinuous large-sized grains, which have grown on top of the smaller grains. Then, for the thin film on the SiO_2_-coated Si wafer, there are angular grains with a larger-than-average grain size of ∼1.28 μm; the distribution of the grains is more uniform, possibly as a result of the lower roughness of the polished SiO_2_ surface (Table S1). This low roughness would present a more uniform surface for film growth, thus assisting film coverage and reducing the likelihood of defects. Such a dense microstructure and better film coverage with larger grains should contribute to high electrical conductivity.^40^

The rate of nucleation at the very beginning of the deposition process depends on the nature of the substrates,^22^ which is one possible reason why the grain shapes and sizes in the lowest layers are different from those of the upper layers in some of the thin films. Some of the thin films, such as those deposited on the ITO-coated glass and mica substrates, show two obvious layers with grains of different sizes and shapes. Because the upper layers are generally discontinuous, carrier transport is expected to be more effective in the lower layers. Chemical reactivity or diffusion at the interface between the thin films and the different substrates could also facilitate the formation of thin films with different morphologies.^19^ The SEM images (Figures 3 and S7) confirm that the substrates have a significant influence on the microstructure of the thin films. EDX data (Figure S8) provide further evidence for the formation of Cu_12_Sb_4_S_13_ in all of the thin films deposited on the different substrates. The different atomic ratios in different regions of the thin films confirm the existence of secondary phases, which may affect charge transport through carrier scattering.

**Figure 4:**
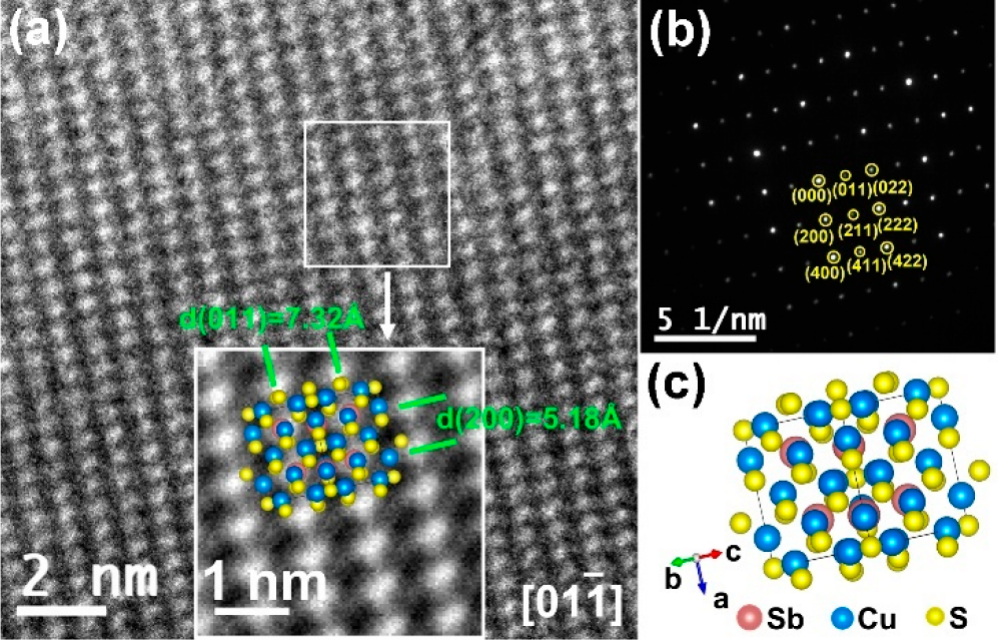
(a) High-resolution transmission electron microscopy (HRTEM) image of Cu_12_Sb_4_S_13_ on the Sb_2_O_3_-coated glass substrate, and an inverse FFT image of the labeled white area (inset). (b) Selected area electron diffraction (SAED) pattern for the area in (a). (c) Ball model of Cu_12_Sb_4_S_13_.

To further investigate the nanostructure of the Cu_12_Sb_4_S_13_ thin films, HRTEM images and SAED patterns for the thin film deposited on the Sb_2_O_3_-coated glass substrate were collected. As illustrated in Figure 4a, the inset inverse fast Fourier transform (FFT) image of the area labeled with a white box clearly reveals that the interplanar spacings of (011) and (200) are 7.32 and 5.18 Å, respectively, which agree well with those for tetrahedrite Cu_12_Sb_4_S_13_ (ICDD: 01-088-0283). The SAED pattern of the area in Figure 4a shows crystal planes of (200), (011), and (211), which are consistent with earlier work.^41^

Next, the Cu_12_Sb_4_S_13_ thin film deposited on the Sb_2_O_3_-coated glass substrate was analyzed by Raman spectroscopy (Figure S9a). A main peak and a minor peak appear at 352 and 325 cm^–1^, respectively, which match well with previous works on Cu_12_Sb_4_S_13_ thin films.^18,42^ The optical bandgap of the Cu_12_Sb_4_S_13_ thin film deposited on the Sb_2_O_3_-coated glass substrate was estimated from the optical absorption spectra using eq 4:^43^
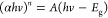
4where α is the absorption coefficient, *hv* is the photon energy, *A* is a constant, and *E*_g_ is the bandgap. As Cu_12_Sb_4_S_13_ is reported to be a direct bandgap semiconductor,^44,45^*n* = 2 was used in the present work. Using the Tauc plot shown in Figure S9b, the bandgap of the Cu_12_Sb_4_S_13_ thin film was estimated to be ∼1.6 eV, which is close to previously reported results.^42,46^

### Thermoelectric Properties

**Figure 5:**
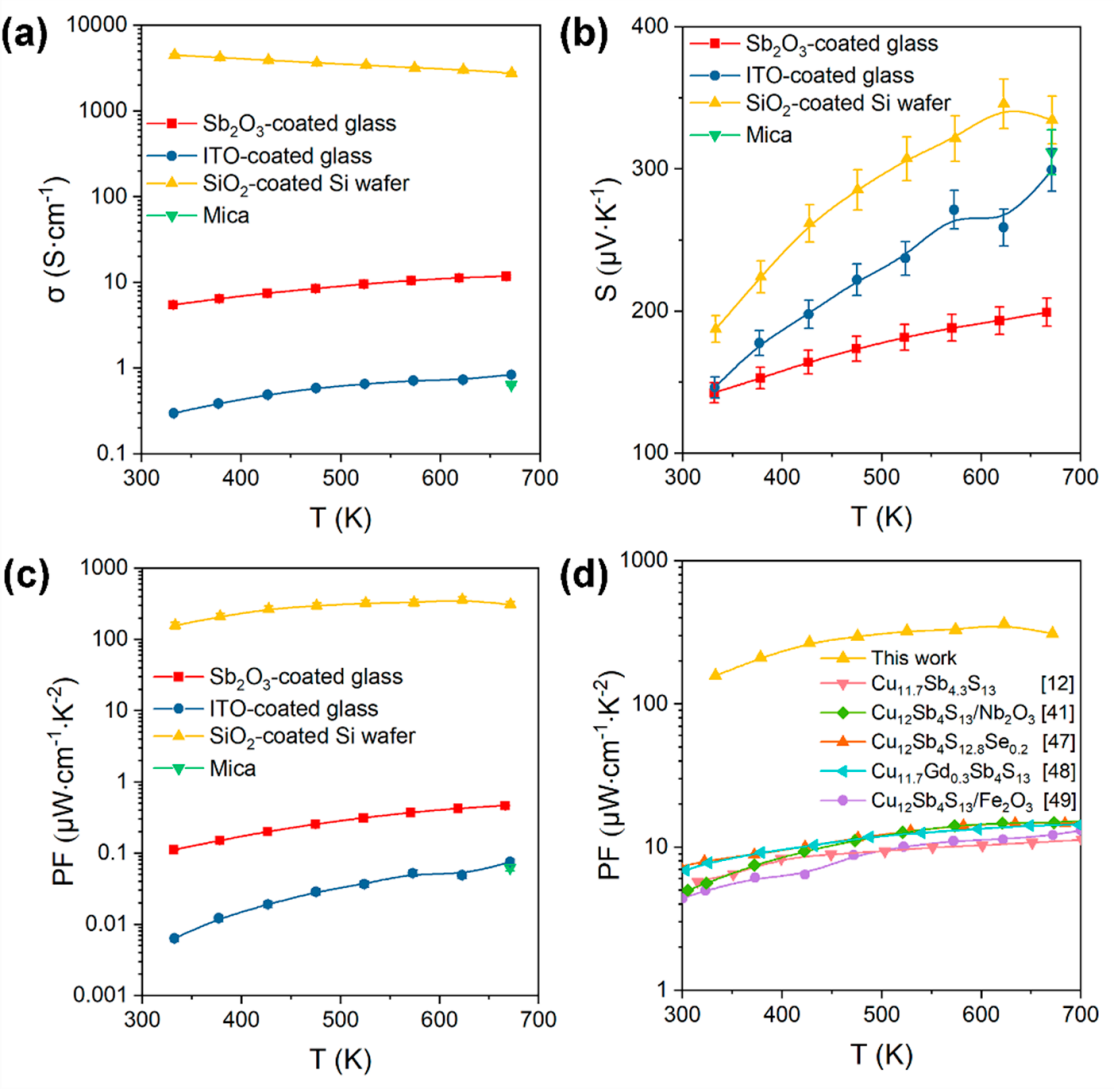
Temperature-dependent (a) electrical conductivity σ, (b) Seebeck coefficient *S*, and (c) power factor PF data for the Cu_12_Sb_4_S_13_ thin films deposited on the following different substrates: glass, Sb_2_O_3_-coated glass, ITO-coated glass, a SiO_2_-coated Si wafer, and mica. The uncertainties in the obtained Seebeck coefficients, electrical conductivities, and power factors were estimated to be 5%, 3%, and 10%, respectively. (d) Comparison of PF in this work to previously published data for Cu_12_Sb_4_S_13_-based bulk materials.^12,41,47−49^

Figure 5 shows the temperature-dependent (325–675 K) in-plane electrical conductivity σ, Seebeck coefficient *S*, and power factor PF data for the tetrahedrite Cu_12_Sb_4_S_13_ thin films deposited on the different substrates. Below 625 K, the electrical conductivities for the Cu_12_Sb_4_S_13_ thin film on the glass substrate and the thin film on mica were too low to be reliably determined, most likely due to poor film coverage (see Figures 3, S7, and S8). Moreover, the impact of secondary phases on the electrical transport properties should not be neglected, as both CuSbS_2_ and Cu_3_SbS_4_ possess lower σ values than Cu_12_Sb_4_S_13_.^12,16^ The electrical conductivity of the film deposited on the substrate with an Sb_2_O_3_ buffer layer (Figure 5a) is much higher (∼10 S cm ^–1^).The XRD and SEM data for this film highlight its superior coverage, reduced impurity phases, and uniform grain structure, all of which enhance carrier transport. The thin film deposited on the ITO-coated glass has a lower σ value than that of the film deposited on the Sb_2_O_3_-coated substrate, despite the high electrical conductivity of ITO and the small impurity phase content that exists in the well-covered thin film. The lower σ value of the thin film on the ITO-coated glass is ascribed to the discontinuous nature of the microstructure of the thin film, which hinders carrier transport. The flake-like grains and smaller-than-average grain size could also contribute to the low electrical conductivity. At the highest measurement temperature (675 K), the thin film on mica achieved a similar electrical conductivity to that of the thin film on the ITO-coated glass. The highest σ value of ∼4000 S cm^–1^ was recorded for the thin film deposited on the SiO_2_-coated Si wafer. It can be inferred that this is a result of this thin film possessing a dense microstructure of large grains, superior film coverage, a higher degree of preferred orientation, and a lower content of secondary phases (Figure 3e). The conductive nature of the Si wafer may also contribute to the high electrical conductivity (Figure 5a).

Data for the Seebeck coefficients of the thin films are presented in Figure 5b. The *S* values in the measured temperature range for the Cu_12_Sb_4_S_13_ thin film deposited on the ITO-coated glass are higher than those for the film deposited on the Sb_2_O_3_-coated glass, suggesting that carrier concentration might dominate the increase of electrical conductivity. However, the thin film deposited on the SiO_2_-coated Si wafer exhibits the highest *S* values across the entire temperature range in spite of its high σ. The simultaneously high Seebeck coefficient and electrical conductivity values of this thin film are expected to have resulted primarily from the enhanced carrier mobility stemming from its more compact microstructure, larger grains, and improved film coverage. Less variation in carrier concentration is anticipated because of the similarity of the compositions of the primary phases of the Cu_12_Sb_4_S_13_ thin films, such as those deposited on the Sb_2_O_3_-coated glass and the SiO_2_-coated Si wafer. Benefiting from a large electrical conductivity value and a nondegraded Seebeck coefficient, the thin film deposited on the SiO_2_-coated Si wafer achieved the highest power factor of ∼362 μW cm^–1^ K^–2^ at 625 K (Figure 5c). This PF value is much larger than those observed for Cu_12_Sb_4_S_13_-based bulk materials reported in the past five years (Figure 5d).^12,41,47−49^

**Figure 6:**
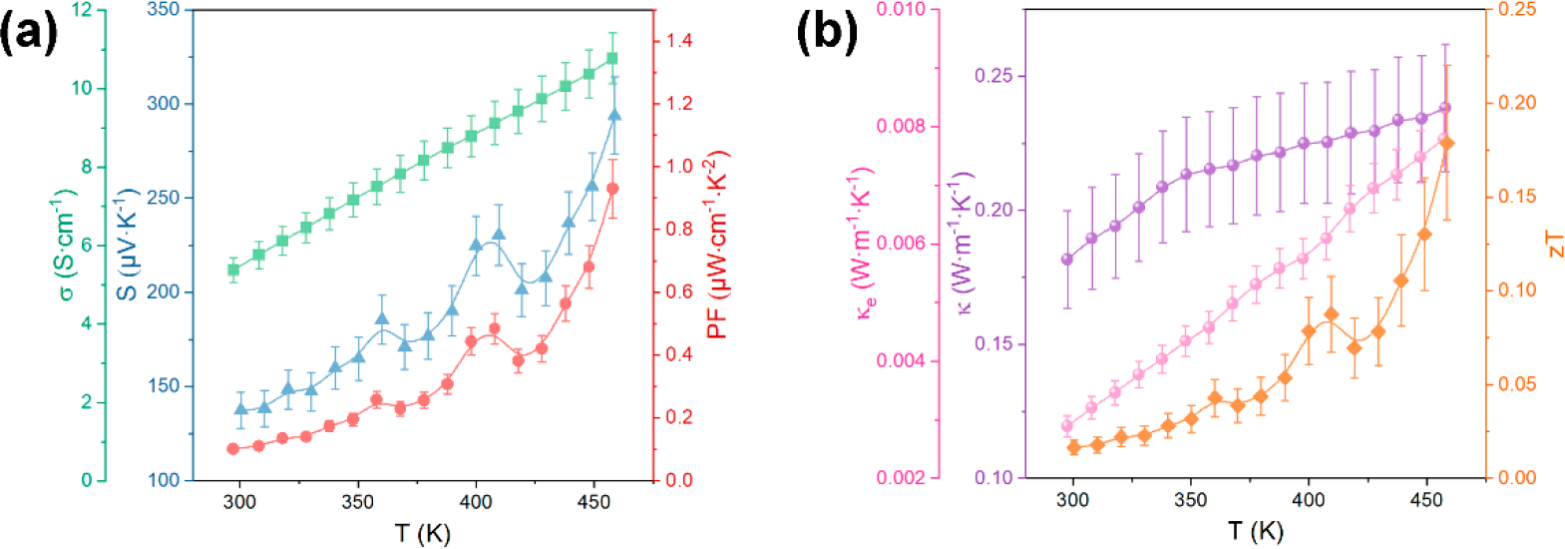
(a) Temperature-dependent Seebeck coefficient *S*, electrical conductivity σ, and power factor PF data, and (b) temperature-dependent thermal conductivity κ, electronic thermal conductivity *κ*_e_, and figure of merit *zT* data for the Cu_12_Sb_4_S_13_ thin film deposited on a TFA test chip with a 300 nm Si_3_N_4_ membrane. The uncertainties in the obtained Seebeck coefficients, electrical conductivities, power factors, thermal conductivities, and *zT* values were estimated to be 7%, 6%, 15%, 10%, and 25%, respectively, according to Linseis et al.^36,37^ and eq 1.

Next, a complete thermoelectric characterization of the Cu_12_Sb_4_S_13_ thin film at temperatures ranging from 300 to 460 K was conducted using a Linseis thin film analyzer (TFA). As shown in Figure 6a, both *S* and σ increased simultaneously with temperature, contributing to the highest PF value of ∼0.93 μW cm^–1^ K^–2^ at 460 K. The analogous trend and similarity in values to those for the thin film deposited on the Sb_2_O_3_-coated glass in the range 300–460 K (Figure 5) prove the reliability of the TFA measurements for the Cu_12_Sb_4_S_13_ thin films. The PF value of the thin film on the test chip obtained by TFA is close to that of the thin film on the Sb_2_O_3_-coated glass and much lower than that of the thin film on the SiO_2_-coated Si wafer; this result is possibly due to the thin film on the test chip having similar grain shapes and sizes to those of the thin film deposited on the Sb_2_O_3_-coated glass (Figures 3b and S11). For the first time, the thermal conductivity κ of the Cu_12_Sb_4_S_13_ thin film is measured, and *zT* is obtained. The calculation of the electronic thermal conductivity *κ*_e_ is shown in the Supporting Information, and the data are presented in Figure 6b. The relatively low κ value of 0.18–0.24 W m^–1^ K^–1^ (Figure 6b) might have benefited from the increased phonon scattering at the film interface^50^ and the intrinsically low thermal conductivity of Cu_12_Sb_4_S_13_.^6,49^ The *zT* value for the Cu_12_Sb_4_S_13_ thin film deposited on the TFA test chip increased with temperature, reaching a maximum value of ∼0.18 at 460 K. We can only speculate that the *zT* value for the Cu_12_Sb_4_S_13_ thin film deposited on the SiO_2_-coated Si wafer (which had a higher PF value) would be much higher. However, the current value of 0.18 at 460 K is comparable to results reported at 460 K for Cu_12_Sb_4_S_13_ bulk materials in recent studies.^47,51,52^ For further enhancing the thermoelectric performance of Cu_12_Sb_4_S_13_ thin films in future research, exploring various doping strategies appears to be a promising avenue. Drawing inspiration from the investigations of polycrystalline Cu_12_Sb_4_S_13_ bulk materials, a variety of dopants (e.g., Sn, Ni, Zn, Gd, etc.^13,48,52^) are worthy of investigation for Cu_12_Sb_4_S_13_ thin films.

**Figure 7:**
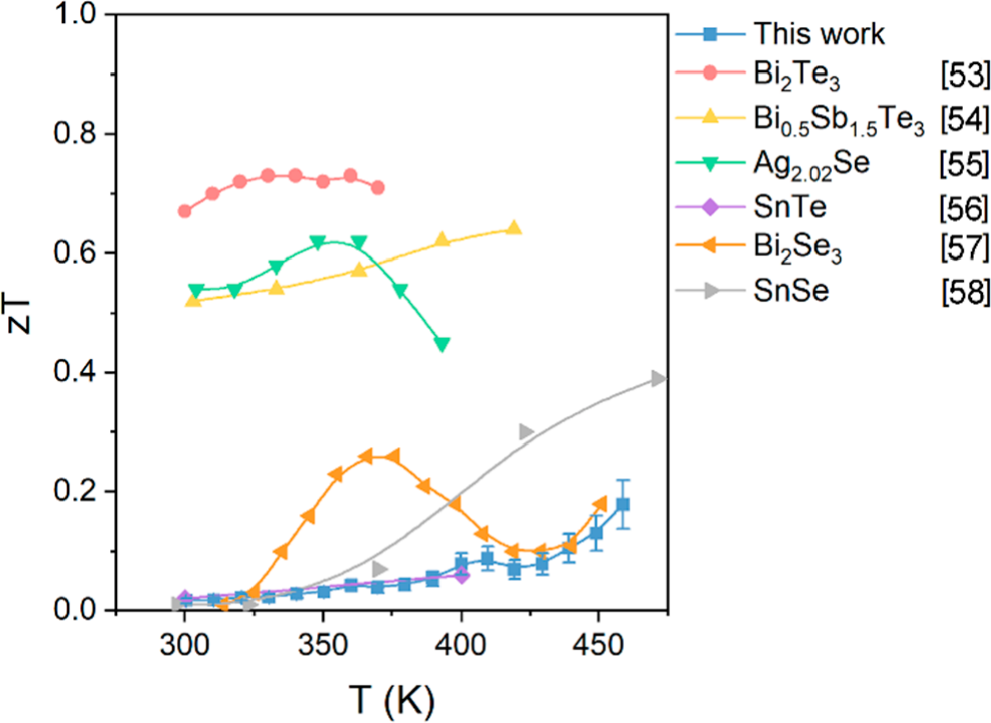
Comparison of *zT* values for the Cu_12_Sb_4_S_13_ thin film on the instrument-dedicated test chip obtained in this work with those for metal chalcogenide thin films reported in the past five years.^53−58^

In Figure 7, the *zT* values for the Cu_12_Sb_4_S_13_ thin film obtained in this work are compared with those for metal chalcogenide thin films reported in the past five years.^53−58^ Our results are comparable to those for Bi_2_Se_3_ above 400 K, those for SnSe below 375 K, and those for SnTe thin films across the whole temperature range. Considering the low cost and easy operation of AACVD, as well as the earth abundance and low toxicity of sulfur when compared to selenium and tellurium, Cu_12_Sb_4_S_13_ thin films obtained via CVD show great potential for manufacturing small-scale, portable thermoelectric devices. Moreover, this study demonstrates that employing suitable substrates or buffer layers can improve the quality of the thin films and enhance their thermoelectric performance, thereby expanding the range of available substrate materials and assisting the development of high-quality, thin-film-based thermoelectric devices.

## Conclusions

In this study, tetrahedrite Cu_12_Sb_4_S_13_ thin films were deposited via AACVD on glass with and without an Sb_2_O_3_ buffer layer, commercially purchased ITO-coated glass, a Si-coated SiO_2_ wafer, and mica. The growth and electrical transport properties of the Cu_12_Sb_4_S_13_ thin films were investigated. It was determined that the quality of the thin film was influenced by the choice of substrate, with notable differences observed in the film coverage, formed impurity phases, preferred orientation, lattice distortions, and grain size and shape. The inclusion of an Sb_2_O_3_ buffer layer between the substrate and the thin film led to a reduction in the impurity phase content and improved coverage uniformity. Notably, the Cu_12_Sb_4_S_13_ thin film deposited on the SiO_2_-coated Si wafer exhibited a dense and compact microstructure, a large grain size, and a lower impurity phase content, which facilitated carrier transport. For the thin film deposited on the SiO_2_-coated Si wafer, a maximum PF of 362 μW cm^–1^ K^–2^ was obtained at 625 K, which is larger than those for most Cu_12_Sb_4_S_13_-based bulk materials reported in the last five years. To the best of our knowledge, the first full thermoelectric characterization of the Cu_12_Sb_4_S_13_ thin film was performed in this work, yielding a maximum *zT* value of ∼0.18 at 460 K. Overall, it was determined that selecting a lattice-matched substrate and using an oxide buffer layer are effective methods for influencing and controlling the thermoelectric properties of tetrahedrite Cu_12_Sb_4_S_13_ thin films.

## Supporting Information Available

Phase diagram of the Cu–Sb–S system, schematic diagram of aerosol-assisted chemical vapor deposition, synthesis details and thermal decomposition analyses for the precursors, crystal structure details and lattice parameters for the substrate materials, SEM images of the Sb_2_O_3_ buffer layer, effects of the various substrates on the Cu_12_Sb_4_S_13_ thin films, Rietveld refinement details, calculated LF values, SEM and EDX data for the Cu_12_Sb_4_S_13_ thin films, Raman spectra, and SEM images of the Cu_12_Sb_4_S_13_ thin film deposited on a TFA test chip (PDF)
