# Supplementary material for: Thermoelectric Performance of Tetrahedrite (Cu12Sb4S13) Thin Films: The Influence of the Substrate and Interlayer
Source: ACS Appl Electron Mater. 2023 Sep 25;6(5):2900–8. doi: 10.1021/acsaelm.3c00909 (PMC11137820; doi:10.1021/acsaelm.3c00909)
Supplement: Supplementary file 1 — el3c00909_si_001.pdf [file el3c00909_si_001.pdf]

Supporting Information

Thermoelectric performance of tetrahedrite ($Cu_{12}Sb_4S_{13}$) thin films: the influence of the substrate and interlayer

Yu Liu [a], Andrey V. Kretinin [a, b], Xiaodong Liu [a], Weichen Xiao [a], David J. Lewis* [a], and Robert Freer* [a]

[a] *Department of Materials, University of Manchester, Oxford Road, Manchester, M13 9PL, UK.*

[b] *National Graphene Institute, University of Manchester, Oxford Road, Manchester, M13 9PL, UK.*

* Corresponding authors: Robert Freer, David J. Lewis; e-mail: robert.freer@manchester.ac.uk and david.lewis-4@manchester.ac.uk

Phase diagram for the Cu-Sb-S system

Abbreviation	Composition
cv	CuS
fcc	$Cu_{2-x}S$
fm	Cu_3SbS_4
td	$Cu_{12}Sb_4S_{13}$
B	Cu_3SbS_3
cstb	$CuSbS_2$
stb	Sb_2S_3
L	Liquid
β	Cu-Sb alloy
Cu_{SS}	Copper solid solution

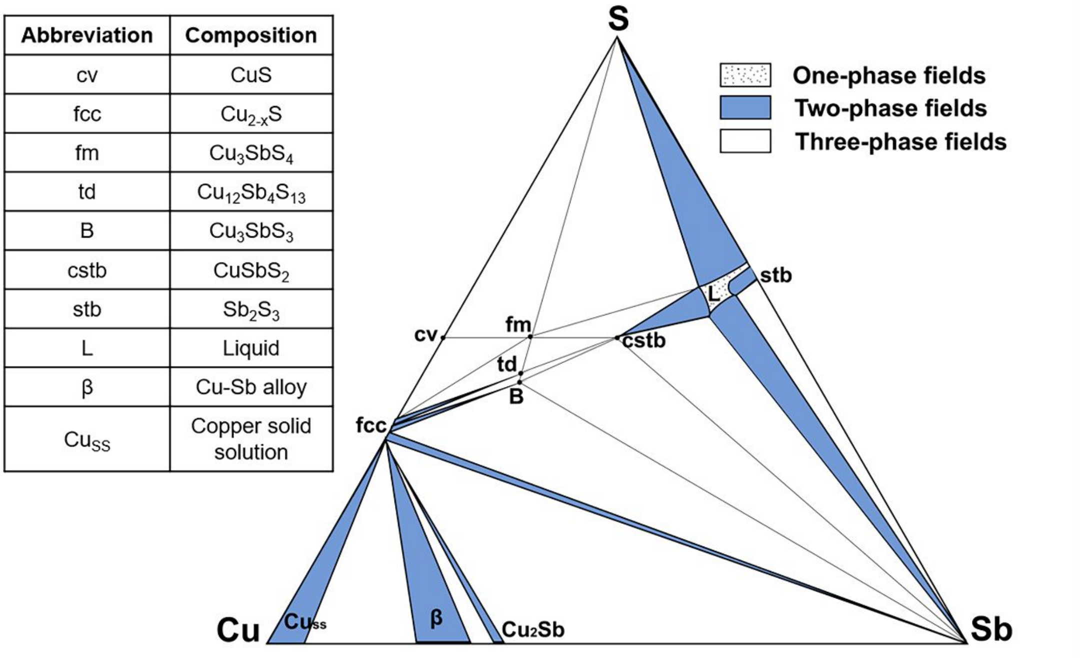

Figure S1. Phase relations in the Cu-Sb-S system at 500 °C; compositions are plotted in atom percent (adapted from the work of Skinner *et al.*).[1]

Aerosol-Assisted Chemical Vapour Deposition (AACVD)

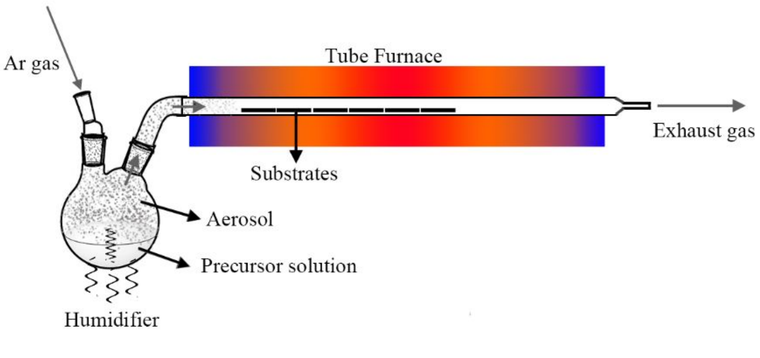

Figure S2. Schematic diagram of aerosol-assisted chemical vapour deposition (AACVD).

Synthesis and thermal decomposition analysis of the precursors

Synthesis of bis(diethyldithiocarbamate)copper(II) $[Cu(S_2CN(C_2H_5)_2)_2]$: 0.06 mol sodium diethyldithiocarbamate trihydrate $[Na(S_2CN(C_2H_5)_2)\cdot 3H_2O]$ (13.5 g) was dissolved in 200 mL methanol. 0.03 mol copper(II) chloride ($CuCl_2$; 4.1 g) was dissolved in 50 mL methanol. A $CuCl_2$ solution was added dropwise to the $Na(S_2CN(C_2H_5)_2)$ solution with stirring. A black precipitate was formed, and after further stirring for 60 min, the product was obtained by filtering, washing (using 60 mL deionized water and 60 mL methanol sequentially), and drying under vacuum at room temperature overnight. Yield: 9.1 g (84 wt%). Elemental analysis: calculated values for $C_{10}H_{20}N_2S_4Cu$: - C, 33.36%; H, 5.60%; N, 7.78%; S, 35.62%; Cu, 17.65%; found - C, 33.26%; H, 5.63%; N, 7.72%; S, 35.63%; Cu, 17. 50%.

Synthesis of tris(diethyldithiocarbamate)antimony(III) $[Sb(S_2CN(C_2H_5)_2)_3]$: 0.06 mol $Na(S_2CN(C_2H_5)_2)$ (13.5 g) and antimony(III) chloride ($SbCl_3$; 4.6g) were fully dissolved in 200 mL methanol and 50 mL methanol, respectively. A $SbCl_3$ solution was added dropwise to the $Na(S_2CN(C_2H_5)_2)$ solution with stirring. The mixture was stirred for 60 min, and then a yellow precipitation was filtered and washed using 60 mL deionized water and 60 mL methanol sequentially. The product was dried under vacuum at room temperature overnight. Yield: 10.2 g (91 wt%). Elemental analysis: calculated values for $C_{15}H_{30}N_3S_6Sb$: - C, 31.80%; H, 5.34%; N, 7.42%; S, 33.96%; Sb, 21.49%; found - C, 31.72%; H, 5.47%; N, 7.36%; S, 33.76%; Sb, 21.77%.

Synthesis of tris(O-ethylxanthato)antimony(III) $[Sb(S_2COC_2H_5)_3]$: 0.06 mol $SbCl_3$ (4.6g) was dissolved in 50ml methanol. The obtained solution was added dropwise to a solution of 0.06 mol (O-ethylxanthato)potassium(I) $[K(S_2COC_2H_5)]$ (9.6 g) in 200ml of methanol. After stirring for 60 min, the mixture was filtered, and the residues were washed with 60 mL deionized water and 60 mL methanol. The

yellow product was dried under a vacuum at room temperature overnight. Yield: 8.2 g (85 wt%). Elemental analysis: calculated values for $C_9H_{15}O_3S_6Sb$: - C, 22.27%; H, 3.11%; O, 9.89%; S, 39.64%; Sb, 25.09%; found - C, 22.46%; H, 3.08%; O, 9.59%; S, 39.92%; Sb, 24.95%.

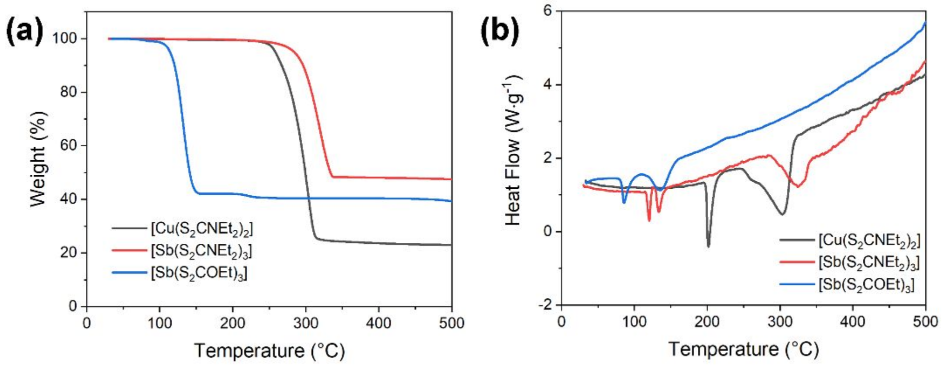

Figure S3. (a) Thermogravimetric analysis (TGA) and (b) differential scanning calorimetry (DSC) of bis(diethyldithiocarbamate)copper(II) [$Cu(S_2CN(C_2H_5)_2)_2$], tris(diethyldithiocarbamate)antimony(III) [$Sb(S_2CN(C_2H_5)_2)_3$], and tris(O-ethylxanthato)antimony(III) [$Sb(S_2COC_2H_5)_3$].

Structure and lattice parameters of the substrate materials

Table S1. Materials used as substrates: surface roughness (root mean square, rms), electrical conductivity (σ), crystal structure, lattice parameters and lattice mismatch f between $Cu_{12}Sb_4S_{13}$ and the substrate.[a]

Substrate	Material at substrate surface	Roughness (nm)	σ (S cm^{-1})	Crystal structure	Lattice parameters (Å)	f (%)
Normal glass	Amorphous glass	1.3	0	N/A	N/A	N/A
Sb_2O_3-coated glass	Sb_2O_3	N/A	0	Cubic	$a = b = c = 11.15$	7.5
ITO-coated glass	ITO	1.8	~6.25-~7.14	Cubic	$a = b = c = 10.09$	2.7
SiO_2 coated Si wafer	Polished SiO_2	0.59	>50	Hexagonal	$a = 5.06$, $b = 5.06$	51.2
					$c = 5.54$	46.6
Mica	Mica (0 0 1)	0.7	0	Monoclinic	$a = 5.19$	50.0
					$b = 9.01$	13.1
					$c = 20.05$	93.3

[a] The rms roughness for commercial glass slides, SiO_2-coated Si wafer, and mica were taken from published data.[2-4] The rms roughness for commercial indium tin oxide (ITO)-coated glass and the electrical conductivity for ITO-coated glass and SiO_2 coated Si wafter were taken from manufacturers data.[5]

SEM of Sb_2O_3 buffer layer

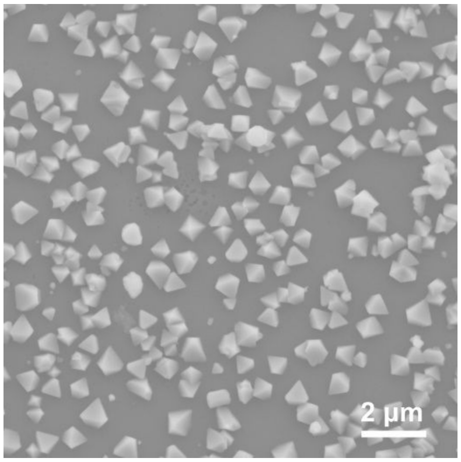

Figure S4. Scanning electron microscopy (SEM) image of Sb_2O_3 buffer layer deposited on glass substrate.

Thin films on different substrates

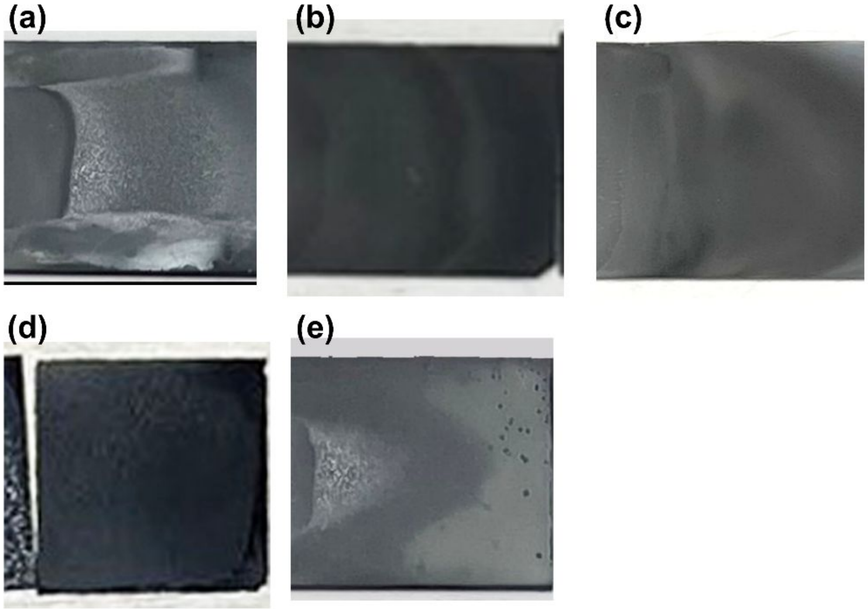

Figure S5. Optical images of $Cu_{12}Sb_4S_{13}$ thin films deposited on different substrates: (a) glass, (b) Sb_2O_3-coated glass, (c) ITO-coated glass, (d) SiO_2-coated Si wafer and (e) mica.

Table S2. Refined lattice parameters for $Cu_{12}Sb_4S_{13}$ films on different substrates and details of impurity phases.

Substrate	Lattice parameter of $Cu_{12}Sb_4S_{13}$ *a* (Å)	Impurity phase	Amount of impurity phase (%)	R_{wp}	GOF
Glass	10.3786(14)	Cu_3SbS_4<br>$Cu_{1.8}S$	13(2)<br>7(2)	3.36	1.92
Sb_2O_3-coated glass	10.3591(7)	Cu_3SbS_4	13(3)	3.13	1.57
ITO-coated glass	10.3501(6)	Cu_3SbS_4	6(1)	2.83	1.60
SiO_2-coated Si wafer	10.3555(9)	Cu_3SbS_4	9(1)	3.27	1.96
Mica	10.3632(9)	$CuSbS_2$	22(2)	3.06	1.69

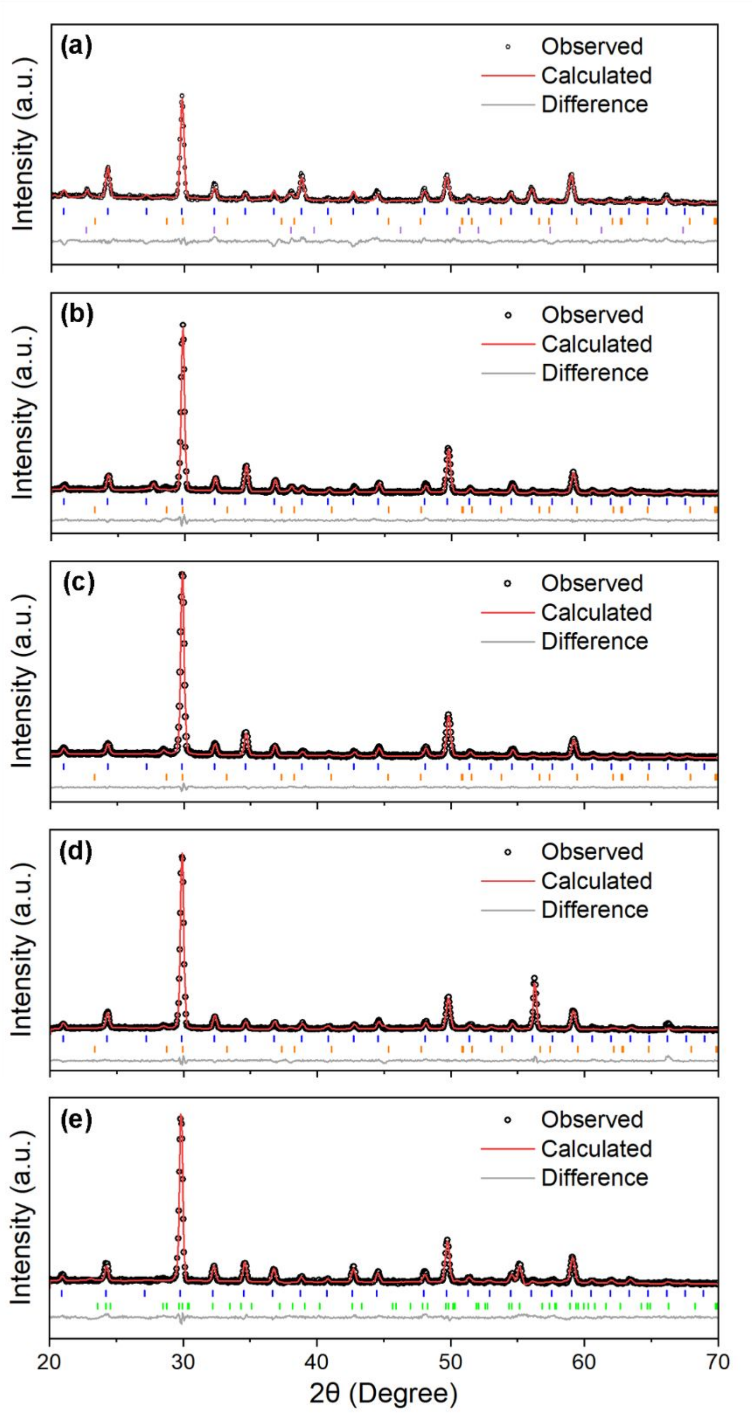

Figure S6. Rietveld refinement of XRD patterns for $Cu_{12}Sb_4S_{13}$ thin films deposited on different substrates: (a) glass, (b) Sb_2O_3-coated glass, (c) ITO-coated glass, (d) SiO_2-coated Si wafer and (e) mica. Bragg reflections for $Cu_{12}Sb_4S_{13}$, Cu_3SbS_4, $CuSbS_2$ and $Cu_{1.8}S$ are represented by blue, orange, green and purple vertical lines, respectively.

Table S3. Lotgering factors (*LF*) for (620) and (2*kl*) planes for $Cu_{12}Sb_4S_{13}$ thin films deposited on different substrates.

Substrate	*LF* (620) (±10%)	*LF* (2*kl*)* (±10%)
Glass	0.027	N/A
Sb_2O_3-coated glass	N/A	0.023
ITO-coated glass	N/A	0.114
SiO_2-coated Si wafer	0.117	0.008
Mica	N/A	N/A

*(2*kl*) represents average values based on (200), (211), (220) and (222) planes

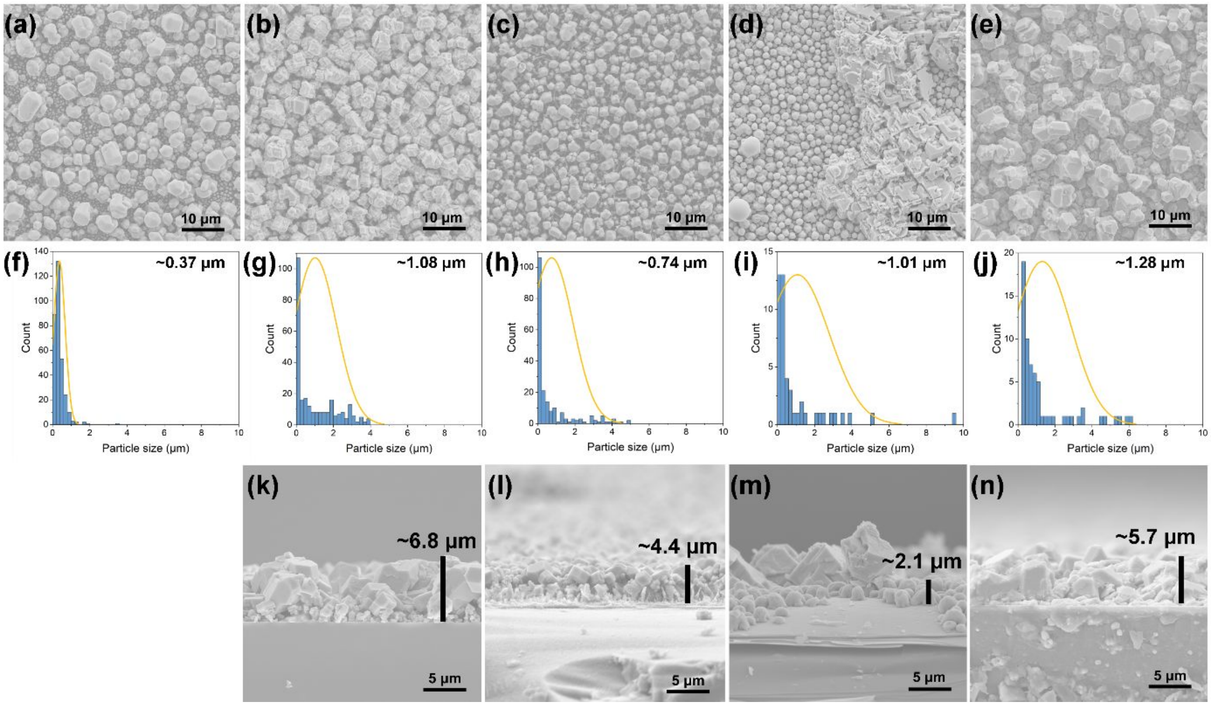

Figure S7. Plan-view, cross-sectional scanning electron microscopy (SEM) images and particle size distributions for $Cu_{12}Sb_4S_{13}$ thin films deposited on different substrates: (a)(f) glass, (b)(g)(k) Sb_2O_3-coated glass, (c)(h)(l) IT-coated glass, (d)(i)(m) SiO_2-coated Si wafer, and (e)(j)(n) mica.

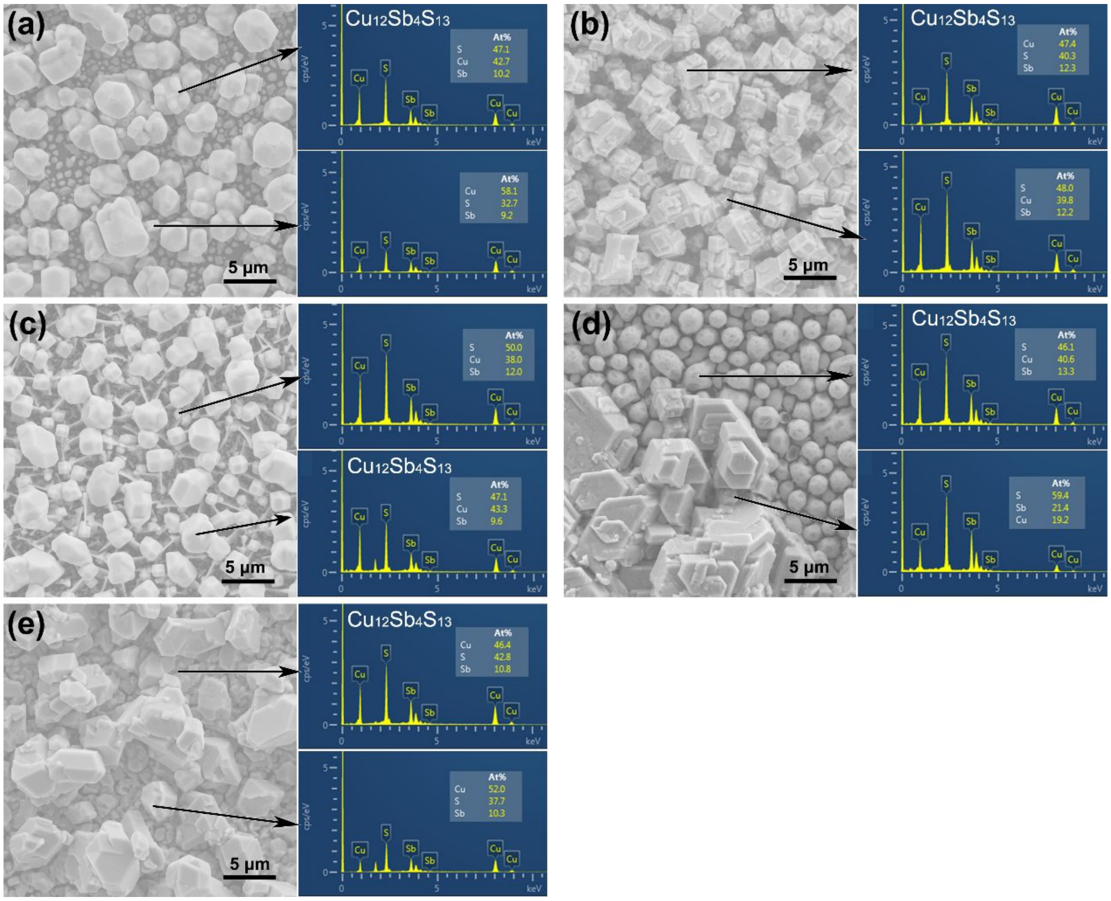

Figure S8. Plan view SEM images and energy-dispersive X-ray spectroscopy (EDX) spectra (and element percentages of Cu, Sb, and S) for $Cu_{12}Sb_4S_{13}$ thin films deposited on different substrates: (a) glass, (b) Sb_2O_3-coated glass, (c) ITO-coated glass, (d) mica, and (e) SiO_2-coated Si wafer.

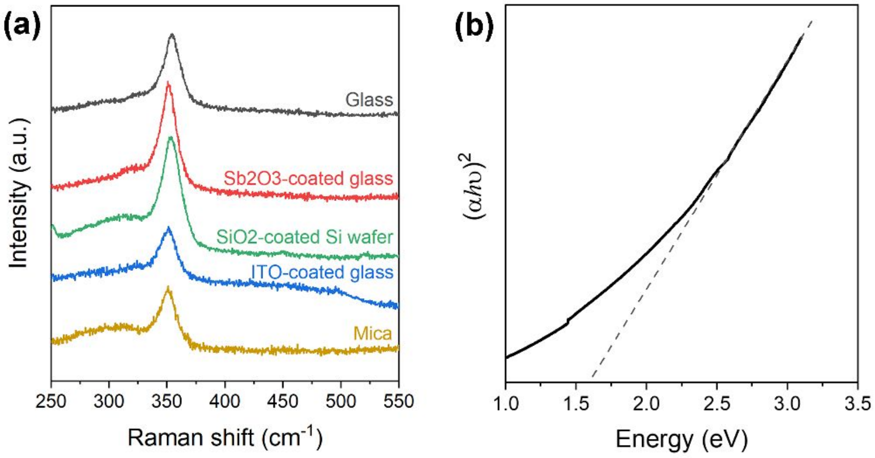

Figure S9. (a) Raman spectra for $Cu_{12}Sb_4S_{13}$ thin film deposited on different substrates and (b) optical absorption spectra for $Cu_{12}Sb_4S_{13}$ thin film deposited on Sb_2O_3-coated glass substrate.

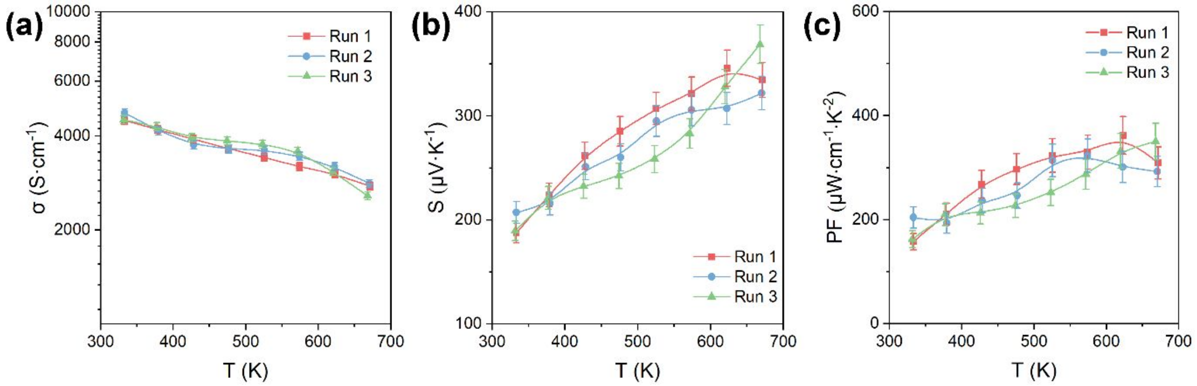

Figure S10. Repeated ZEM measurements for temperature-dependent (a) electrical conductivity σ, (b) Seebeck coefficient *S*, and (c) power factor *PF* for a $Cu_{12}Sb_4S_{13}$ thin film deposited on SiO_2 coated Si wafer.

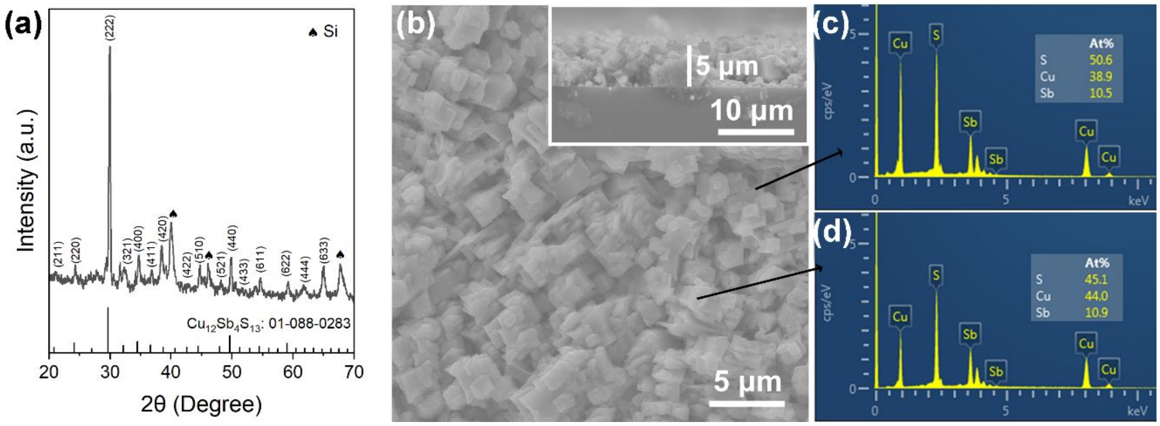

Figure S11. (a) X-ray diffraction (XRD) pattern, (b) plan-view and (inset) cross-sectional scanning electron microscopy (SEM) images and (c, d) energy dispersive X-ray spectroscopy (EDX) spectra of the $Cu_{12}Sb_4S_{13}$ thin film deposited on the TFA test chip with a 300 nm Si_3N_4 membrane. The test chip consists of a silicon matrix and Si_3N_4 membrane; the thin film was deposited on the membrane area. Due to the small area of the membrane, XRD peaks corresponding to Si were also detected.

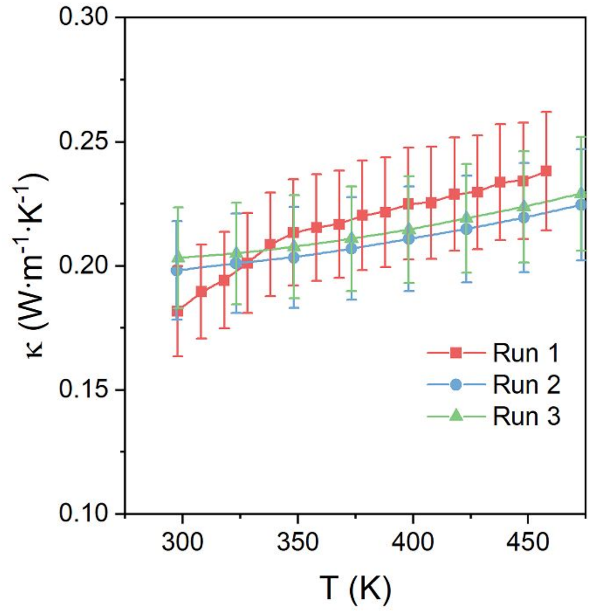

Figure S12. Repeat TFA measurements for temperature-dependent thermal conductivity of the $Cu_{12}Sb_4S_{13}$ thin film deposited on TFA test chip with 300 nm Si_3N_4 membrane.

The electronic thermal conductivity κ_e was calculated via the Wiedemann-Franz law $\kappa_e = \sigma LT$,[6] where σ is electrical conductivity, L is Lorenz factor and T is temperature. L can be calculated using the equation:[7] $L = \left(1.5 + \exp\left[-\frac{|S|}{116}\right]\right) \times 10^{-8}\,\mathrm{V}^2\,\mathrm{K}^{-2}$. The calculated Lorenz factor is shown in Figure S11, and the calculated electronic thermal conductivity is shown in Figure 6b.

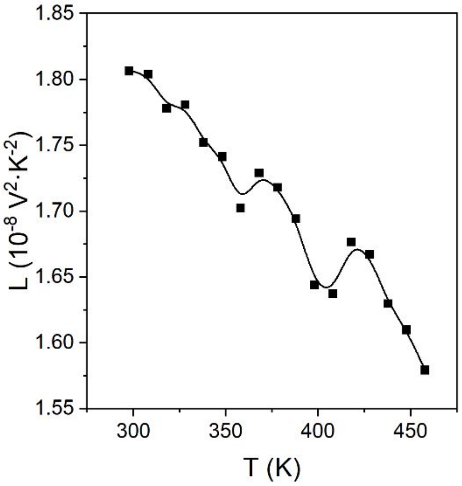

Figure S13. Temperature-dependent Lorenz factor of the $Cu_{12}Sb_4S_{13}$ thin film deposited on the test chip with Si_3N_4 membrane.

References:

(1) Skinner, B. J.; Luce, F. D.; Makovicky, E. Studies of the sulfosalts of copper III; Phases and phase relations in the system Cu-Sb-S. *Econ. Geol.* **1972**, *67* (7), 924-938.

(2) Matsumae, T.; Koehler, A. D.; Suga, T.; Hobart, K. D. A scalable clean graphene transfer process using polymethylglutarimide as a support scaffold. *J. Electrochem. Soc.* **2016**, *163* (6), E159-E161.

(3) Yu, J.; Yu, J. C.; Cheng, B.; Zhao, X.; Zheng, Z.; Li, A. Atomic force microscopic studies of porous TiO_2 thin films prepared by the sol-gel method. *J. Sol-Gel Sci. Technol.* **2002**, *24*, 229-240.

(4) Bhowmik, D.; Karmakar, P. Tailoring and investigation of surface chemical nature of virgin and ion beam modified muscovite mica. *Surf. Interface Anal.* **2019**, *51* (6), 667-673.

(5) *Ossila Ltd*. https://www.ossila.com/products/ito-glass-substrates-unpatterned (accessed 2023 16 June).

(6) Snyder, G. J.; Toberer, E. S. Complex thermoelectric materials. *Nat. Mater.* **2008**, *7*, 105-114.

(7) Kim, H.-S.; Gibbs, Z. M.; Tang, Y.; Wang, H.; Snyder, G. J. Characterization of Lorenz number with Seebeck coefficient measurement. *APL Mater.* **2015**, *3* (4), 041506.
